# Stage-of-change Assessment Predicts Short-term Treatment Engagement for Opioid Use Disorder Patients Initiated on Buprenorphine

**DOI:** 10.5811/westjem.2022.3.53197

**Published:** 2022-06-29

**Authors:** Quentin Reuter, Gregory L. Larkin, Michael Dubé, Suman Vellanki, Amanda Dos Santos, Jamie McKinnon, Nicholas Jouriles, David Seaberg

**Affiliations:** * Summa Health System, Department of Emergency Medicine, Akron, Ohio; † Summa Health System, Department of Psychiatry, Akron, Ohio; ‡ US Acute Care Solutions, Canton, Ohio

## Abstract

**Introduction:**

The emergency department (ED) is an effective setting for initiating medication for opioid use disorder (MOUD); however, predicting who will remain in treatment remains a central challenge. We hypothesize that baseline stage-of-change (SOC) assessment is associated with short-term treatment retention outcomes.

**Methods:**

This is a longitudinal cohort study of all patients enrolled in an ED MOUD program over 12 months. Eligible and willing patients were treated with buprenorphine at baseline and had addiction medicine specialist follow-up arranged. Treatment retention at 30 and 90 days was determined by review of the Prescription Drug Monitoring Program. We used uni- and multivariate logistic regression to evaluate associations between patient variables and treatment retention at 30 and 90 days.

**Results:**

From June 2018–May 2019, 279 patients were enrolled in the ED MOUD program. Of those patients 151 (54.1%) and 120 (43.0%) remained engaged in MOUD treatment at 30 and 90 days, respectively. The odds of treatment adherence at 30 days were significantly higher for those with advanced SOC (preparation/action/maintenance) compared to those presenting with limited SOC (pre-contemplation/contemplation) (60.0% vs 40.8%; odds ratio 2.18; 95% confidence interval 1.15 to 4.1; P <0.05). At 30 days, multivariate logistic regression determined that advanced SOC, age >40, having medical insurance, and being employed were significant predictors of continued treatment adherence. At 90 days, advanced SOC, non-White race, age > 40, and having insurance were all significantly associated with higher likelihood of treatment engagement.

**Conclusion:**

Greater stage-of-change was significantly associated with MOUD treatment retention at 30 and 90 days post index ED visit.

## INTRODUCTION

The opioid epidemic currently claims more than 180 lives per day in the United States, with deaths topping over 46,000 in 2018.^1^–^6^ Due to the mounting societal, economic, and health consequences, the opioid epidemic was declared a national public health emergency in 2017.^7^ Despite national awareness for this growing emergency, the majority of patients with opioid use disorder (OUD) don’t have access to addiction medicine services.^8^

For patients with OUD, the emergency department (ED) represents a critical access point for receiving medical care and, thus, an important opportunity to reach OUD patients. Medication for opioid use disorder (MOUD) has been shown to decrease mortality, reduce overdoses, increase treatment retention, and decrease the costs associated with addressing the opioid epidemic.^9^–^15^ Further research, including work done by the authors, has described the implementation and short-term results of other ED-initiated MOUD programs.^16^–^19^ Despite these efforts, treatment retention remains a significant challenge to successfully initiating MOUD from the ED. For example, D’Onofrio et al reported encouraging 30-day treatment retention outcomes in a well-resourced academic medical center program, but these rates fell to less than 50% at 6 and 12 months.^20^ Other ED-initiated MOUD programs show an even greater decline in treatment retention after the initial 30-day follow-up period.^16^–^19^

Predicting who will remain in MOUD treatment continues to be a vexing challenge for the medical system. Various patient characteristics have been reported to predict MOUD treatment success, albeit many of these associations are inconsistent across the literature. Younger age, male gender, Black ethnicity, concomi-tant substance use disorders (SUD), hepatitis C, previous opioid overdoses, homelessness, unemployment, and criminal activity have all been associated with higher rates of treatment failure.^21^–^28^ Despite these reports, no data exists regarding predictive characteristics for MOUD treatment success in ED populations.

The transtheoretical model, also known as stage-of-change (SOC), was developed decades ago by Prochaska and DiClemente to better assess a patient’s willingness to address high-risk behaviors and has shown predictive value across numerous settings including stress management, medication adherence, psychotherapy, weight management, and SUD.^29^–^36^ The SOC is assessed by addiction medicine professionals via in-depth interviews discerning a patient’s desire for recovery, level of self-awareness, and exhibited actions toward addiction recovery. Assessment of a patient’s readiness for recovery from SUD using SOC has never been studied in ED OUD patients.

Against this background, the objective of this study was to examine the association of SOC to predict treatment retention at 30 and 90 days for patients enrolled in a community hospital-based ED-initiated MOUD treatment program.

## METHODS

### Study Design

This is a prospective observational study of patients enrolled in MOUD from the ED. As MOUD is viewed as standard of care, this study was reviewed and received an exemption from the institutional review board.

### Study Setting and Population

Participants were enrolled at one community hospital with over 33,000 annual ED visits over a 12-month period (June 2018–May 2019). Patients >18 years old were eligible for enrollment if they had OUD and a positive clinical opioid withdrawal score (COWS) of *≥* 8 as measured by an ED nurse and verified by an emergency physician. Patients were identified either by self-referral or by physician/nursing identification.

**Population Health Research Capsule**

What do we already know about this issue?

*Emergency department-initiated medication for opioid use disorder (MOUD) is a safe and effective treatment modality for opioid use disorder*.

What was the research question?

*Does stage-of-change (SOC) assessment predict 30- and 90-day treatment retention in ED patients started on MOUD?*

What was the major finding of the study?

*Patients with advanced SOC were 2.18 times more likely to be in treatment at 30 and 90 days compared to MOUD patients with limited pre-contemplation*.

How does this improve population health?

*Assessment for SOC can help identify ED MOUD patients at high risk for treatment failure and thus guide more aggressive interventions*.

Patients who were already established in a SUD treatment program, including those who tested positive for methadone or who had a history of methadone use on their Ohio Prescription Drug Monitoring Program (PDMP) report, were excluded and referred back to their treatment program. Pregnant patients were not enrolled but rather were transferred to high-risk maternal fetal medicine for further management. We also excluded all patients requiring admission to the hospital. Patients who did not clinically qualify for MOUD were evaluated by the addiction care coordinator (ACC) and provided a referral to a clinically appropriate treatment option.

Data including patient demographics, SOC level, confidence in ability to quit, and COWS score were collected and documented in the medical record by the ACC during the ED visit. Patients’ SOC was assessed via in-depth ACC interviews with them to discern their readiness for change. For example, patients in denial or who were unaware of their addiction would be considered to be in the “pre-contemplation” phase. Patients experimenting with small behavioral changes and collecting information about recovery services are generally deemed to be in “preparation” phase. Finally, individuals demonstrating direct actions, such as seeking out addiction medicine services, were considered to be in the “action” phase.

### Study Protocol

All patients who met inclusion criteria were evaluated and medically cleared by the emergency physician or advance practice provider. Initial Emergency Medical Treatment and Active Labor Act screening was similar to that performed on any other ED patient but additionally, per protocol, included complete blood counts, comprehensive metabolic panel including liver function tests, ethanol level, pregnancy test if indicated, and urine drug screening.

After the patient completed the medical screening exam, they were seen by the ACC, a nurse with specialized training in addiction medicine. The ACC conducted a thorough interview with eligible patients and evaluated each criterion of the American Society of Addiction Medicine (ASAM) six-dimension assessment. The ASAM six-dimension assessment evaluates a patient’s acute intoxication and/or withdrawal potential, biomedical conditions, emotion/behavioral/cognitive conditions, readiness to change, relapse potential, and living environment. Each of the six dimensions is assigned a risk rating to help identify the greatest barriers to recovery and formulate a treatment plan. After the patient was first introduced to the idea of addiction recovery, the ACC then provided the patient with personalized feedback, attempted to enhance patient motivation, and finally negotiated and advised on a specific treatment plan. The ACC used motivational interviewing to help patients explore their understanding, desire, and barriers to positive behavior change.

The SOC was also assessed via an extensive interview to elicit the patient’s desire for recovery, motivations behind seeking treatment, and actions planned or already taken to rehabilitate. Based on these factors, a patient was assigned a specific SOC. If the patient was eligible, the ACC directly connected the patient with an addiction medicine referral and reviewed and ensured the patient’s eligibility for addiction services and insurance clearance, as well as helped arrange transportation. If a patient’s COWS score was <8, the ACC still evaluated the patient, provided addiction medicine education and counseling. The patient was instructed to return to the ED later that day or the next for induction into the program.

If a patient was deemed eligible, and consented to enrollment in the MOUD program, treatment with buprenorphine was initiated during the index ED visit. Treatment consisted of buprenorphine 8 milligrams (mg)/2mg for one dose followed by two hours of observation. If their repeat COWS score at two hours was still >8, the patient received a second dose of buprenorphine. Once the patient’s withdrawal symptoms were controlled, they were observed for approximately 1.5 hours and discharged.

Urgent outpatient addiction medicine follow-up was arranged by the ACC. This follow-up included office-based opioid treatment for buprenorphine management and an intensive outpatient program for counseling.

The MOUD program was staffed by four ACC nurses with a total of 2.2 full-time equivalents (FTE). Our program coordinator, at 0.25 FTE, was also required to organize training, monitor data collection, and complete administrative tasks. Education for the ACCs consisted of 18 hours of instruction covering topics in addiction, stigma, MOUD treatment modalities, SBIRT (Screening, Brief Intervention and Referral to Treatment), harm reduction, and peer support. In total, costs for salary and benefits for administrative and ACC personnel, as well as their training, was $246,616/year.

### Measures

Baseline patient demographics including age, gender, race/ethnicity, and medical and/or psychiatric comorbidities at the time of MOUD induction were collected via chart review. At the index visit the ACC assessed and recorded the patient’s COWS, highest level of education, insurance status, employment type, SUD type, tobacco use, pregnancy status, baseline SOC, marital status, financial and legal concerns, residence type, and spirituality. These data elements were extracted via chart review. Using the PDMP, we assessed treatment retention at 30 and 90 days. Treatment retention was defined as patients receiving regular buprenorphine/naloxone prescriptions at 30 and 90 days from index ED visit date.

Regarding chart review methodology, the abstractors included four physicians involved in the evaluation of the MOUD project. These abstractors were not blinded to the hypothesis. Prior to data extraction, the abstractors were adequately trained in the chart review methodology, including data element identification. Standardized abstraction forms were used, and any missing data was identified as such. We conducted duplicate chart review assessments to help ensure accurate data extraction.

### Data Analysis

The primary outcome of interest was the association of patient baseline SOC and engagement in treatment measured at 30 and 90 days post index ED visit. Secondary outcomes included associations of patient demographic factors and treatment retention at 30 and 90 days. We determined univariate differences of proportion using Fisher’s exact test. Candidate covariates were screened at the *P* **≤** 0.1 level of significance. Multivariate models were run on the field of candidate variables using backward stepwise logistic regression to evaluate the strength of association between patient variables and treatment retention at 30 and 90 days. Variables were retained in the model if significant at the *P* < 0.05 level. We conducted all analyses using SPSS Statistics version 27 (IBM Corp., Armonk, NY).

## RESULTS

From June 2018–May 2019 the ACCs evaluated patients during 691 visits, screened 571 unique patients, and enrolled 279 patients in the ED MOUD program (Figure). Of the patients enrolled, 196 (70.3%) were male, and ages ranged from 18–74, with 193 (69.2%) being younger that 40 years old. Ethnicity mirrored that of the surrounding community and was largely White: 253 (90.7%). Only 85 (31.8%) had education beyond high school or GED, 148 (53.6%) were unemployed, and 54 (19.6%) were self-pay. A total of 180 (70%) reported financial concerns, 42 (16.1%) were married, 171 (66.5%) reported having children, and 46 (16.5%) were undomiciled (Table). The average ED length of stay for the 279 patients enrolled in the MOUD program was 283 minutes.

At 30 days post ED enrollment, 151 (54.1%) of the patients enrolled in the MOUD program were engaged in treatment (Figure), The odds of treatment adherence at 30 days were significantly higher for those with advanced SOC (preparation/action/maintenance) compared to those presenting with limited SOC (precontemplation/ contemplation) (60.0% vs 40.8%; odds ratio [OR] 2.18; 95% confidence interval [CI] 1.15 to 4.1; *P* <0.05). Multivariate logistic regression determined that younger age (<40 years; OR 0.52; 95% CI, 0.28–0.98; *P* <0.01) and being uninsured (OR 0.29; 95% CI 0.14–0.58; *P* <0.01) were significant risk factors for disengagement at 30 days while both advanced SOC (OR 2.4; 95% CI 1.2–4.7; *P* <0.05) and baseline employment (OR 1.9; 95% CI 1.06–3.4; *P* <0.01) were significant predictors of continued treatment adherence.

At 90 days post enrollment, 120 (43.0%) patients were engaged in treatment (Figure 1). Advanced SOC (OR 2.65; 95% CI 1.3–5.5; *P* <0.01), non-White race (OR 2.7; 95% CI 1.02–7.1; *P* <0.05), age > 40 (OR 2.2; 95% CI 1.2–3.9; *P* <0.01), and having insurance (OR 2.56; 95% CI,1.26–5.2; *P* <0.01) were all retained in the multivariate logistic regression model and associated with significantly higher likelihood of treatment engagement at 90 days. Self-reported confidence in ability to quit, gender, having children, mental health comorbidities, and presenting to ED with an overdose were not significantly associated with staying in treatment at either 30 or 90 days.

## DISCUSSION

This longitudinal cohort study of patients enrolled in an ED-initiated MOUD program describes 30- and 90-day treatment retention outcomes. We also describe the factors associated with treatment retention– importantly advanced SOC. Our patient population was generally poor and underinsured with over half being unemployed, nearly 20% being self-pay, and 70% reporting financial concerns. Demographic factors associated with treatment retention included age >40, having medical insurance, and being employed. Interestingly, advanced SOC was also associated with higher levels of treatment retention at 30 and 90 days, while patient-reported level of confidence in ability to quit was not.

The ED represents our healthcare system’s safety net and cares for patients with SUD on a frequent basis. Broadly deploying evidence-based treatment strategies in EDs, such as MOUD, is a vital strategy for combating the opioid epidemic. Prior to the implementation of the MOUD program, our ED clinicians had relatively little to offer OUD patients that directly addressed their underlying addiction. While anecdotal, we believe that by using MOUD, we have begun to rebuild trust between OUD patients and the medical system. A once generally negative relationship between OUD patients and our ED staff has been replaced with a hopeful rapport, confident that recovery for these patients is a distinct possibility. This therapeutic relationship con-tinues to grow and we believe will lead to long-term sustained recovery for many of our OUD patients in the surrounding community.

The predictive utility of SOC assessment in patients with SUD has produced inconclusive results.^35^–^41^ Regarding ED patients with SUD specifically, we found no literature to date that describes the utility of assessing SOC in this population. Of the mixed results in the literature, numerous publications have provided positive evidence for SOC assessment in SUD patients. Henderson et al found the ability of SOC to predict treatment outcomes in patients receiving buprenorphine for OUD was nearly statistically significant.^35^ In adolescents admitted to a 28-day residential treatment program for alcohol, marijuana, cocaine, and amphetamine use disorder, Callaghan et al reported less advanced SOC to be predictive of treatment dropouts.^38^ DiClemente found SOC was predictive of alcohol use disorder recovery outcomes at 12 months.^42^ Our work further validates the utility of SOC assessment in SUD patients, specifically in ED patients with OUD.

Other research has been unsuccessful in reproducing these positive findings relating SOC to SUD treatment outcomes. Siegal et al failed to show a relationship between SOC and treatment retention in veterans with cocaine use disorder undergoing a month of residential treatment.^43^ Similarly, Gossop et al found no association between initial SOC and self-reported opioid and stimulant use at one year post induction in a large patient cohort of 1075 patients with SUD.^40^

Our work differs from these studies in numerous ways. Our patient population consists of community ED patients with OUD, rather than other primary SUDs in various other clinical settings. It is possible the ED OUD population represents a particularly motivated group of patients urgently seeking recovery services, and thus SOC assessment at their initial evaluation is more predictive of treatment retention. Furthermore, our intervention consisted of outpatient treatment with agonist therapy rather than inpatient and residential interventions. It may be more useful to measure SOC in the outpatient arena as compared to inpatient programs, which use more intensive treatment interventions that may override the impact of a patient’s initial readiness to change. Moreover, our outcomes are not reported by patients but rather defined as patients receiving agonist therapy from an addiction medicine clinician, strengthening the validity that SOC has predictive utility. Finally, it would be difficult to show a link between a single SOC assessment and outcomes 12 months later, as the Gossop paper attempted to evaluate.

Our results suggest that evaluating SOC at baseline may help identify patients more and less likely to remain in treatment and thus could offer innovative opportunities to tailor care to this population. More intensive resources, closer monitoring, and more frequent interactions with medical care could be used to improve treatment retention in patients with less advanced SOC. Conversely, patients with advanced SOC may represent a patient population truly committed to overcoming the challenges of OUD and may warrant additional resources to address social and monetary barriers to recovery. While more work is needed to verify the utility of SOC in this setting, we believe our findings represent an exciting and novel avenue for delivering personalized care to ED patients with OUD.

Interestingly, SOC was predictive of a patient’s ability to stay in recovery, while a patient’s self-reported confidence in their ability to quit was not. An antonym of false self-assurance is self-effacement or humility. Post et al describe the importance of patient humility on their ability to successfully navigate recovery in the Alcoholics Anonymous (AA) program.^44^ Step One in AA is “We admitted we were powerless over alcohol—that our lives had become unmanageable.” One’s ability to accept some level of powerlessness and fully embrace the coming struggle and need for assistance on their journey to sobriety represents a self-aware and open mindset that can optimize one’s chance for success. Future research is needed to confirm whether a denial of powerlessness or over-confidence in one’s ability to quit represents a lack of humility and thus leads to a higher risk of recidivism.

## LIMITATIONS

There are various limitations to our study. First, our work is a prospective observational study, and as such there was no control group or randomization. Our study population was from a single community hospital and may not be generalizable to other clinical settings. The ACCs are specially trained nursing staff with an expertise in addiction medicine and were vital to the success of the program. While it may not be feasible for every community hospital to create ACC positions, assessing SOC in OUD patients is a relatively simple process and may be automated as well. We used four different data extractors and didn’t complete duplicate chart reviews for the vast majority of the patients. To help ensure consistent data collection, however, we used a standardized data extraction form and performed a limited number of duplicate chart reviews. Using the PDMP may have undercounted the number of patients still engaged in treatment as it may not have revealed individuals receiving treatment from in-patient and day programs.

We were significantly limited by the number of patients in groups “pre-contemplation” and “maintenance” (2 and 10 patients, respectively). Thus, we needed to dichotomize the SOC assessment to conduct a meaningful statistical analysis, which was a novel SOC application and may not be generalizable. Finally, given the limited data on the factors associated with MOUD retention in ED populations, we decided to evaluate all variables collected by the ACC at time of enrollment using our multivariate model rather than examining independent variables with a plausible role in a sequential process. We felt this would prevent us from cherry-picking potential variables and result in an assessment of our specific patient population that was relatively free from bias.

## CONCLUSION

Predicting who will remain in treatment is a central challenge in caring for ED patients initiated on medications for opioid use disorder. Advanced state of change was significantly associated with MOUD treatment retention at 30 and 90 days, while self-reported confidence in one’s ability to quit was not associated with treatment adherence. Future work should validate the SOC risk metric in this patient population and determine methods to help nudge patients from pre-contemplation/contemplation to action. Capturing SOC data may allow for more tailored treatment of patients at highest risk of disengagement and overall non-adherence.

## Figures and Tables

**Figure:**
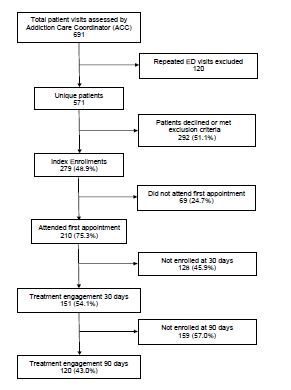
Patient enrollment and retention in a medication for opioid use disorder program at 30 and 90 days. *ED*, emergency department.

**Table:** 30-day and 3-month treatment retention rates related to various patient factors.

Patient variables		Enrollment status over time	

	N (%)	Retention at 30 days n (%)	Retention at 90 Days n (%)
Gender			
Male	196 (70.3)	101(51.5)	80(40.8)
Female	83 (29.7)	50 (60.2)	40(48.2)
Age (years) Mean (SEM): 36.7 (0.66) Range: 18–67			
Mean age (SEM) Enrolled: yes vs no		38.9(0.95) v. 34(0.85)**	39.7(1.1) v. 34.3(0.77)**
< 40 years	193 (69.2)	92 (47.7)**	72 (37.3)**
≥ 40 years	86 (30.8)	59 (68.6)	48 (55.8)
Race			
White	253 (90.7)	134 (53)	105(41.5)*
Other	26 (9.3)	17 (65.4)	15 (57.7)
Education			
Lower (High school/GED or less)	182 (68.2)	95 (52.2)	71 (39)
>Post high school education	85 (31.8)	52 (61.2)	45 (52.9)
Employment			
Unemployed	148 (53.6)	68 (45.9)**	56 (37.8)
Employed	128 (46.4)	82 (64.1)	63 (49.2)
Insurance status			
Insured	225 (80.6)	133 (59.1)	107 (47.6)
Self-pay	54 (19.4)	18 (33.3)**	13 (24.1)**
Psychiatric comorbidities			
Depression	76 (27.2)	44 (57.9)	38 (50)
Anxiety/PTSD	74 (26.5)	42 (56.8)	35 (47.3)
Bipolar	31 (11.1)	16 (51.6)	14 (45.2)
Schizophrenia	10 (3.6)	3 (30)	3 (30)
History of suicide attempt	5 (1.8)	1 (20)	1 (20)
Any psych comorbidity	118 (42.3)	8 (57.6)	56 (47.5)
No psych comorbidity	161 (57.7)	83 (51.6)	64 (39.8)
Social demographics			
Married	42 (16.1)	29 (69)	20 (47.6)
Unmarried	219 (83.9)	117 (53.4)	94 (42.9)
Children	171 (66.5)	102 (59.6)	76(44.4)
No children	86 (33)	43 (50)	36 (41.9)
Legal concerns	78 (30.4)	36 (46.2)*	31 (39.7)
No legal concerns	179 (69.6)	108 (60.3)	81 (45.3)
Financial concerns	180 (70)	95 (52.8)	70 (38.9)*
No financial concerns	77 (30.0)	49 (63.6)	42 (54.5)
Homeless	46 (16.5)	16 (34.8)**	16 (34.8)
Domiciled	233 (83.5)	135 (57.9)	104 (44.6)
Confidence in ability to quit			
Less confidence	97 (39.6)	52 (53.6)	41 (42.3)
Extremely confidence (10/10)	148 (60.4)	86 (58.1)	66 (44.6)
Stage of change			
Precontemplation	2 (1)	0	0
Contemplation	47 (18.5)	20 (42.6)*	13 (27.7)*
Preparation	99 (39.0)	56 (56.6)	49 (49.5)
Action	96 (37.8	60 (62.5)	44 (45.8)
Maintenance	10 (3.9)	7 (70)	5 (50)
Limited stage of change: Pre-contemplation/contemplation	49 (19.3)	20 (40.8)*	13 (26.5)**
Advanced stage of change Preparation/action/maintenance	205 (80.7)	123 (60)	98 (47.8)
TOTAL	279 (100)	151 (54.1)	120 (43)

* = P <0.05;

** = P <0.01.

*SEM*, standard error of mean; *GED*, general education development; *PTSD*, post-traumatic stress disorder; psych, psychiatric.

## References

[1] Centers for Disease Control and Prevention (2015). National vital statistics system mortality data.

[2] Centers for Disease Control and Prevention (2015). Vital signs: today’s heroin epidemic.

[3] Centers for Disease Control and Prevention (2015). QuickStats: Rates of deaths from drug poisoning and drug poisoning involving opioid analgesics — United States, 1999–2013.

[4] Scholl L, Seth P, Kariisa M (2018). Drug and opioid-involved overdose deaths - United States, 2013–2017. MMWR Morb Mortal Wkly Rep.

[5] National Institute on Drug Abuse Overdose death rates.

[6] Wilson N, Kariisa M, Seth P (2020). Drug and opioid-involved overdose deaths - United States, 2017–2018. MMWR Morb Mortal Wkly Rep.

[7] Mercia D (2017). Trump declares opioid epidemic national public health emergency.

[8] Volkow N, Koob G, McLellan A (2016). Neurobiologic advances from the brain disease model of addiction. N Engl J Med.

[9] Mattick R, Breen C, Kimber J (2014). Buprenorphine maintenance versus placebo or methadone maintenance for opioid dependence. Cochrane Database Syst Rev.

[10] Amato L, Minozzi S, Davoli M (2011). Psychosocial combined with agonist maintenance treatments versus agonist maintenance treatments alone for treatment of opioid dependence. Cochrane Database Syst Rev.

[11] Sordo L, Barrio G, Bravo M (2017). Mortality risk during and after opioid substitution treatment: systematic review and meta-analysis of cohort studies. BMJ.

[12] Schwartz R, Gryczynski J, O’Grady K (2013). Opioid agonist treatments and heroin overdose deaths in Baltimore, Maryland, 1995–2009. Am J Public Health.

[13] Kimber J, Copeland L, Hickman M (2010). Survival and cessation in injecting drug users: prospective observational study of outcomes and effect of opiate substitution treatment. BMJ.

[14] Pierce M, Bird S, Hickman M (2016). Impact of treatment for opioid dependence on fatal drug-related poisoning: a national cohort study in England. Addiction.

[15] Corsi K (2010). Opiate substitute treatment is associated with increased overall survival among injecting drug users. Evid Based Ment Health.

[16] Bogan C, Jennings L, Haynes L (2020). Implementation of emergency department-initiated buprenorphine for opioid use disorder in a rural southern state. J Subst Abuse Treat.

[17] Hu T, Snider-Adler M, Nijmeh L (2019). Buprenorphine/naloxone induction in a Canadian emergency department with rapid access to community-based addictions providers. CJEM.

[18] Kaucher K, Caruso E, Sungar G (2020). Evaluation of an emergency department buprenorphine induction and medication-assisted treatment referral program. Am J Emerg Med.

[19] Reuter Q, Smith G, McKinnon J (2020). Successful medication for opioid use disorder (MOUD) program at a community hospital emergency department. Acad Emerg Med.

[20] D’Onofrio G, Chawarski M, O’Connor P (2017). Emergency department-initiated buprenorphine for opioid dependence with continuation in primary care: outcomes during and after intervention. J Gen Intern Med.

[21] O’Connor A, Cousins G, Durand L (2020). Retention of patients in opioid substitution treatment: a systematic review. PLoS One.

[22] Manhapra A, Agbese E, Leslie D (2018). Three-year retention in buprenorphine treatment for opioid use disorder among privately insured adults. Psychiatr Serv.

[23] Manhapra A, Petrakis I, Rosenheck R (2017). Three-year retention in buprenorphine treatment for opioid use disorder nationally in the Veterans Health Administration. Am J Addict.

[24] Hser Y, Saxon A, Huang D (2014). Treatment retention among patients randomized to buprenorphine/naloxone compared to methadone in a multi-site trial. Addiction.

[25] Weinstein Z, Kim H, Cheng D (2017). Long-term retention in office based opioid treatment with buprenorphine. J Subst Abuse Treat.

[26] Samples H, Williams A, Olfson M (2018). Risk factors for discontinuation of buprenorphine treatment for opioid use disorders in a multi-state sample of Medicaid enrollees. J Subst Abuse Treat.

[27] Kelly S, O’Grady K, Mitchell S (2011). Predictors of methadone treatment retention from a multi-site study: a survival analysis. Drug Alcohol Depend.

[28] Heidebrecht F, MacLeod M, Dawkins L (2018). Predictors of heroin abstinence in opiate substitution therapy in heroin-only users and dual users of heroin and crack. Addict Behav.

[29] Evers K, Prochaska J, Johnson J (2006). A randomized clinical trial of a population- and transtheoretical model-based stress-management intervention. Health Psychol.

[30] Johnson S, Driskell M, Johnson J (2006). Transtheoretical model intervention for adherence to lipid-lowering drugs. Dis Manag.

[31] Johnson S, Driskell M, Johnson J (2006). Efficacy of a transtheoretical model-based expert system for antihypertensive adherence. Dis Manag.

[32] Johnson S, Paiva A, Cummins C (2008). Transtheoretical model-based multiple behavior intervention for weight management: effectiveness on a population basis. Prev Med.

[33] Levesque D, Van Marter D, Schneider R (2011). Randomized trial of a computer-tailored intervention for patients with depression. Am J Health Promot.

[34] Velicer W, Redding C, Sun X (2007). Demographic variables, smoking variables, and outcome across five studies. Health Psychol.

[35] Henderson M, Saules K, Galen L (2004). The predictive validity of the University of Rhode Island change assessment questionnaire in a heroin-addicted polysubstance abuse sample. Psychol Addict Behav.

[36] Norcross J, Krebs P, Prochaska J (2011). Stages of change. J Clin Psychol.

[37] Serafini K, Shipley L, Stewart D (2016). Motivation and substance use outcomes among adolescents in a school-based intervention. Addict Behav.

[38] Callaghan R, Hathaway A, Cunningham J (2005). Does stage-of-change predict dropout in a culturally diverse sample of adolescents admitted to inpatient substance-abuse treatment? A test of the transtheoretical model. Addict Behav.

[39] Belding M, Iguchi M, Lamb R (1997). Stages and processes of change as predictors of drug use among methadone maintenance patients. Exp Clin Psychopharmacol.

[40] Gossop M, Stewart D, Marsden J (2007). Readiness for change and drug use outcomes after treatment. Addiction.

[41] Cady M, Winters K, Jordan D (1996). Motivation to change as a predictor of treatment outcome for adolescent substance abusers. Journal of Child & Adolescent Substance Abuse.

[42] Carbonari J, DiClemente C (2000). Using transtheoretical model profiles to differentiate levels of alcohol abstinence success. J Consult Clin Psychol.

[43] Siegal H, Li L, Rapp R (2001). Measuring readiness for change among crack cocaine users: a descriptive analysis. Subst Use Misuse.

[44] Post S, Pagano M, Lee M (2016). Humility and 12-step recovery: a prolegomenon for the empirical investigation of a cardinal virtue in Alcoholics Anonymous. Alcohol Treat Q.

